# Associations between body fat variability and later onset of cardiovascular disease risk factors

**DOI:** 10.1371/journal.pone.0175057

**Published:** 2017-04-03

**Authors:** Yuki Saito, Osamu Takahashi, Hiroko Arioka, Daiki Kobayashi

**Affiliations:** 1 Division of General Internal Medicine, Department of Medicine, St. Luke’s International Hospital, Tokyo, Japan; 2 Center for Clinical Epidemiology, St. Luke's Life Science Institute, Tokyo, Japan; McMaster University, CANADA

## Abstract

**Objective:**

There is current debate regarding whether body weight variability is associated with cardiovascular events. Recently, high body fat percentage (BF%) has been shown to be a cardiovascular risk factor. We therefore hypothesized that BF% variability would present a stronger cardiovascular risk than body weight variability.

**Methods:**

A single-center retrospective cohort study of medical check-up examinees aged 20 years or older at baseline (2005) was performed. Examinees were followed in 2007, 2009, and 2013–2014. BF% variability in 2005, 2007 and 2009 was calculated as the root-mean square error (RMSE) using a simple linear regression model. Multiple logistic regression models estimated the association between BF%-RMSE and new diagnoses of cardiovascular risk factors occurring between the 2009 and 2013–2014 visits.

**Results:**

In total, 11,281 participants (mean age: 51.3 years old, 48.8% were male) were included in this study. The average BF%-RMSE of our subjects was 0.63, and the average BMI-RMSE was 0.24. The high BF%-RMSE group (76-100^th^ percentile) had a higher incidence of hypertension and a lower incidence of diabetes mellitus than the low BF%-RMSE group (1-25^th^ percentile). This tendency was particularly evident in male participants. BMI-RMSE was not associated with any cardiovascular risks in our study.

**Conclusions:**

This study indicates that body fat variability has contrasting effects on cardiovascular risk factors, while body weight variability has no significant effects.

## Introduction

Homeostasis dysregulation is a risk factor for several diseases and for cardiovascular events in particular. For example, blood pressure variability is an independent cardiovascular risk factor regardless of average blood pressure [1–2]. Similarly, variability in fasting glucose and in cholesterol are associated with future cardiovascular events [3–5].

Although the mechanisms underlying the association between homeostasis dysregulation and subsequent cardiovascular disease have not been fully understood, oxidative stress may play a key role, especially in terms of blood pressure and glucose variability [6–9].

In terms of obesity, some previous studies have reported that body weight variability is a risk factor of all-cause mortality and fatal cardiovascular events [10–11], while others have suggested that this association is controversial [12–13]. In the Framingham population study, those with the highest body weight variability had a relative risk ratio of 1.93 in males and 1.55 in females for coronary heart disease-related mortality [10]. This association could partially be explained by weight gain [13], high body mass index (BMI) [14], smoking [15], mental status [16] and low-grade systemic inflammation [17].

Recently, body fat percentage (BF%) has been shown to be a risk factor of mortality and cardiovascular diseases, independent of BMI [18–19], and to be more useful in detecting early stage accumulation of cardiovascular disease risk [20]. Many other studies have provided supportive evidence that BMI and BF% are not analogous [21] and that a high BF% is useful for detecting normal-weight obesity [22–23].

These findings led us hypothesize that body fat variability is a stronger risk factor of cardiovascular events than body weight variability. Some reports have assessed the changes in body fat distribution in weight cyclers [24–25], but no previous studies have focused on the variability in body fat itself. As BMI can be influenced by either increased fatness or greater muscle mass [26], BF% variability may be more appropriate than BMI variability in examining associations with later cardiovascular events.

This study aimed to evaluate the association between BF% variability and cardiovascular risk factors, namely hypertension, dyslipidemia, and diabetes mellitus.

## Method

### Study design

This study aims to evaluate the relationship between fluctuations in BF% and later development of cardiovascular risk factors. In this study, we evaluated BF% fluctuation in the 2005, 2007, and 2009 fiscal years and its association with cardiovascular risk factors (hypertension, dyslipidemia, diabetes mellitus) newly diagnosed between the 2009 and 2013–2014 visits. The institutional review board of St. Luke’s International Hospital Ethics Committee approved all aspects of this study.

### Study participants (Study population)

Our retrospective cohort included individuals aged 20 years or older who received a medical check-up at the Center for Preventive Medicine at St. Luke’s International Hospital, Tokyo, Japan. This check-up program aims to promote health through early detection of cancer, chronic disease, or related risk factors. Employees of various companies, local government organizations in Tokyo and their affiliates comprise approximately 80% of the participants. The other 20% of the participants independently registered for the program at the center.

### Data collection

All data were extracted automatically from the patient’s electronic chart. Participants' demographic characteristics, including alcohol consumption habits, current and previous smoking status, and marital status; medical histories, such as hypertension, dyslipidemia, diabetes mellitus and medication use for these diseases; and family history of cardiovascular and cerebrovascular events in second-degree relatives were obtained from a questionnaire. Weight and height were measured using a body fat meter, BF-220 (Tanita, Japan). Body fat percentage (BF%) and total body fat mass (FM) were also measured using the same machine. It is a digital electronic body composition analyzer used widely in previous studies [27–31], and bioelectrical impedance analysis is used to measure body fat in this machine. Bioelectrical impedance analysis may not be as accurate as dual energy X-ray absorptiometry [32], but its validity in epidemiological studies was reported in previous studies [33–34]. Blood tests related to cardiovascular risk factors, including fasting glucose, hemoglobin A1c (HbA1c), low-density lipoprotein cholesterol (LDL-CHO), high-density lipoprotein cholesterol (HDL-CHO), and triglycerides (TG), were measured.

To evaluate the magnitude of BF% variability, we used the root-mean square error (RMSE) applying a simple linear regression model. Each individual’s BF% values in 2005, 2007, and 2009 were regressed on years from the first visit. In brief, the BF%-slope represents the trend of direction, and the BF%-RMSE, which is the standard deviation around this slope, represents the BF% fluctuation magnitude in this model [13,16–17]. We divided participants into 4 groups according to their BF%-RMSE percentile (Group 1: 0-25^th^ percentile, Group 2: 26-50^th^ percentile, Group 3: 51-75^th^ percentile and Group 4: 76-100^th^ percentile). The same method was applied to BMI. The coefficient of variance is another parameter to describe fluctuation, however a person with a slight and steady weight gain may have the same coefficient of variance as a person with large weight gain and loss [35]. We chose RMSE, because RMSE can be a more sensitivity measure of instability regardless of the overall trend [17].

### Outcomes

Our primary outcomes were newly diagnosed clinical cardiovascular risk factors, including hypertension, diabetes and dyslipidemia, during the follow-up period. In this analysis, hypertension was defined as (1) systolic blood pressure ≥ 140 mmHg or diastolic blood pressure ≥ 90 mmHg or (2) a positive response to having hypertension in the self-reported questionnaire. Diabetes mellitus was defined as (1) HbA1c ≥ 6.5% and fasting glucose ≥ 126 mg/dl or (2) a positive response to having diabetes mellitus in the self-reported questionnaire. Dyslipidemia was defined as (1) LDL-CHO ≥ 140 mg/dl, HDL-CHO < 40 mg/dl or TG ≥ 150 mg/dl or (2) a positive response to having dyslipidemia in the self-reported questionnaire.

### Statistical analysis

All statistical analyses were conducted using STATA software version 11 (STATA Corp., Texas, USA). Chi-square test was used to analyze cross-tabulated data, and one-way ANOVA or Kruskal-Wallis test was used to compare the means of continuous data in the univariate analyses of participants' baseline characteristics. Stratification was applied by gender. Ninety-five percent confidence intervals (95% CI) were calculated using normal approximation methods. We constructed multiple logistic regression models for newly diagnosed diseases indicating cardiovascular risk and calculated the odds-ratios and 95% CIs. Inclusion of the variables in the models were based on existing knowledge of cardiovascular risk factors. Alcohol consumption [36], smoking status [37], exercise [38–39], marital status [40–41], family history of cerebrovascular accidents (CVA) and acute coronary syndrome (ACS) [42–43], and medication use [44–45] were previously reported to affect cardiovascular risks. To show the robustness of our results, we next conducted two different sensitivity analyses. In the first analysis, we performed logistic regression using FM, and the index of FM over height squared (FM [kg] / (height [m])^2^), instead of BF%. In the second analysis, we constructed two additional models adjusting for various subsets of the covariates.

## Results

### Baseline characteristics of subjects

A total of 11,317 individuals received medical check-ups at all visits. Thirty-six patients with missing data (mean age: 57.8 years, 50% were women) were excluded, for a final study sample of 11,281 participants. Individuals who submitted a document refusing participation in the clinical study were planned to be excluded, but no patients submitted this document.

The mean age of our subjects was 51.3 years old (standard deviation (SD) 11.0), and 5,502 (48.8%) were male. Additionally, 25,504 did not attend the follow-up medical check-ups in 2007 or 2009. Table 1 shows a comparison of characteristics between those who completed this study and those did not.

**Table 1:** Characteristics’ comparison between those who completed this study and those didn’t.

	Our subjects (*n* = 11,281)	Other 2005 visitors (*n* = 25,504)
Age, mean, years (SD)	51.3(11.0)	48.4(12.4)
Male, *n*(%)	5,502(48.8)	13,118(51.4)
Alcohol abuse, *n*(%)	4,867(43.1)	11,507(45.1)
Smoking status		
current smoker, *n*(%)	1,561(13.8)	4,915(19.3)
ex-smoker, *n*(%)	2,688(23.8)	5,904(23.2)
Exercise		
everyday, *n*(%)	1,334(11.8)	2,619(10.3)
3–5 days/week, *n*(%)	1,978(17.5)	3,818(15.0)
1–2 days/week, *n*(%)	4,285(38.0)	9,494(37.2)
less than 1 day/week, *n*(%)	3,684(32.7)	9,572(37.5)
Married, *n*(%)	8,468(75.1)	18,494(72.5)
Family history of CVA, ACS*, *n*(%)	2,428(21.5)	5,182(20.3)
Medication use, *n*(%)	1589(14.1)	3012(11.8)
BMI, mean(SD)	22.5(3.2)	22.5(3.3)
BF%, mean, %(SD)	23.8(5.7)	23.7(5.8)

CVA, cerebrovascular accidents; ACS, acute coronary syndrome; BMI, body mass index; BF%, body fat percentage

*: Family history of CVA, ACS included family history of myocardial infarction, angina, stroke, brain hemorrhage, and subarachnoid hemorrhage in second degree relatives.

Table 2 shows the baseline characteristics by BF%-RMSE. Those with a higher BF%-RMSE included a higher percentage of male and smoking subjects, and had a higher BMI and BF%. The prevalence of hypertension and diabetes mellitus in 2009 was significantly higher in the high BF%-RMSE groups. BF%-slope was negative in all groups.

**Table 2:** Baseline characteristics of participants.

variables	BF%-RMSE					*p* values
	Group 1 (*n* = 2,620)	Group 2 (*n* = 2,851)	Group 3 (*n* = 3,044)	Group 4 (*n* = 2,766)	Total (*n* = 11,281)	
	(0-25^th^ percentile)	(26-50^th^ percentile)	(51-75^th^ percentile)	(76-100^th^ percentile)		
Age, mean, years (SD)	51.3 (10.9)	51.7 (11.2)	51.5 (10.9)	50.6 (11.2)	51.3 (11.0)	**<0.01**
Male, *n*(%)	1,210 (46.2)	1,326 (46.5)	1,561 (51.3)	1,405 (50.8)	5,502 (48.8)	**<0.01**
Alcohol abuse, *n*(%)	1,091 (41.6)	1,236 (43.4)	1,339 (44.0)	1,201 (43.4)	4,867 (43.1)	0.33
Smoking status						**0.02**
current smoker, *n*(%)	326 (12.4)	376 (13.2)	438 (14.4)	421 (15.2)	1,561 (13.8)	
ex-smoker, *n*(%)	615 (23.5)	664 (23.3)	758 (24.9)	651 (23.5)	2,688 (23.8)	
Exercise						0.07
everyday, *n*(%)	315 (12.0)	349 (12.2)	362 (11.9)	308 (11.1)	1,334 (11.8)	
3–5 days/week, *n*(%)	489 (18.7)	497 (17.4)	518 (17.0)	474 (17.1)	1,978 (17.5)	
1–2 days/week, *n*(%)	963 (36.8)	1,108 (38.9)	1,200 (39.4)	1,014 (36.7)	4,285 (38.0)	
less than 1 day/week, *n*(%)	853 (32.6)	897 (31.5)	964 (31.7)	970 (35.1)	3,684 (32.7)	
Married, *n*(%)	1,965 (75.0)	2,173 (76.2)	2,306 (75.8)	2,024 (73.2)	8,468 (75.1)	**0.04**
Family history of CVA, ACS*, *n*(%)	549 (21.0)	612 (21.5)	681 (22.4)	586 (21.2)	2,428 (21.5)	0.57
Medication use, *n*(%)	347(13.2)	391(13.7)	437(14.4)	414(15.0)	1,589(14.1)	0.28
Hypertension in 2009, *n*(%)	553 (21.1)	679 (23.8)	747 (24.5)	674 (24.4)	2,653 (23.5)	**0.01**
Dyslipidemia in 2009, *n*(%)	1,032 (39.4)	1,129 (39.6)	1,270 (41.7)	1,144 (41.4)	4,575 (40.6)	0.17
Diabetes Mellitus in 2009, *n*(%)	122 (4.7)	138 (4.8)	145 (4.8)	172 (6.2)	577 (5.1)	**0.03**
BMI, mean(SD)	22.0 (3.1)	22.1 (3.1)	22.5 (3.1)	23.3 (3.5)	22.5 (3.2)	**<0.01**
BF%, mean, %(SD)	23.0 (5.3)	23.2 (5.4)	23.7 (5.5)	25.4 (6.4)	23.8 (5.7)	**<0.01**
BF%-slope, mean(SD)	-0.10 (1.13)	-0.15 (1.15)	-0.18 (1.22)	-0.42 (1.67)	-0.21 (1.31)	**<0.01**

CVA, cerebrovascular accidents; ACS, acute coronary syndrome; BMI, body mass index; BF%, body fat percentage

*: Family history of CVA, ACS included family history of myocardial infarction, angina, stroke, brain hemorrhage, and subarachnoid hemorrhage in second degree relatives.

### Results of the multivariate logistic regression analysis

**Fig 1:**
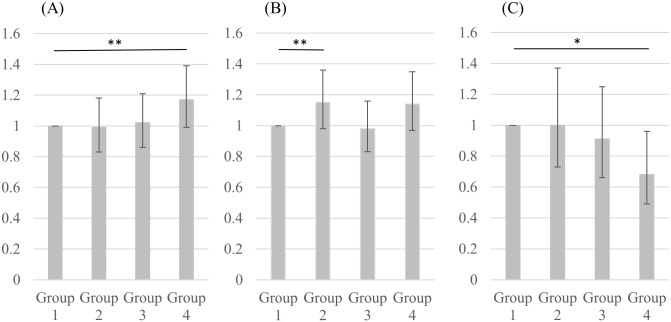
Odds ratios of cardiovascular risk factors in multivariate logistic regression analysis. Hypertension (B) Dyslipidemia (C) Diabetes Mellitus. Group 1 is the reference group. *: p<0.05, **: p<0.10.

We then performed a multivariate logistic regression of the primary end points: hypertension, dyslipidemia, and diabetes mellitus (upper half of Table 3). The odds ratio of the incidence of hypertension in the BF%-RMSE Group 4 was 1.17 (95%CI = 0.99–1.39; *p* = 0.07). Although not significant, there was a steady increase in the development of hypertension as BF%-RMSE rose (Fig 1A). Conversely, the development of diabetes mellitus decreased as BF%-RMSE increased (Fig 1C). The development of diabetes in the BF%-RMSE Group 4 was significantly lower than in Group 1 (OR = 0.68; 95%CI = 0.49–0.96; *p* = 0.03). There was no clear tendency in the incidence of dyslipidemia (Fig 1B).

**Table 3:** Associations between BF%-RMSE/BMI-RMSE and the newly diagnosed cardiovascular risk factors in the multivariate logistic regression analysis.

	Odds ratio (95% confidence interval)			
	Group 1	Group 2	Group 3	Group 4
	(0-25^th^ percentile)	(26-50^th^ percentile)	(51-75^th^ percentile)	(76-100^th^ percentile)
BF%-RMSE^†^				
Hypertension^††^	Reference	0.99 (0.83–1.18)	1.02 (0.86–1.21)	1.17 (0.99–1.39)**
Dyslipidemia^§§^	Reference	1.15 (0.98–1.36)**	0.98 (0.83–1.16)	1.14 (0.97–1.35)
Diabetes Mellitus***	Reference	1.00 (0.73–1.37)	0.91 (0.66–1.25)	0.68 (0.49–0.96)*
BMI-RMSE^§^				
Hypertension^††^	Reference	1.10 (0.93–1.31)	1.12 (0.95–1.32)	1.11 (0.93–1.32)
Dyslipidemia^§§^	Reference	0.93 (0.79–1.10)	1.04 (0.89–1.21)	1.01 (0.85–1.19)
Diabetes Mellitus***	Reference	0.86 (0.62–1.17)	0.74 (0.54–1.00)**	0.79 (0.58–1.08)

*: p<0.05,

**: p<0.10

^†^: Sample size in each BF%-RMSE group Group1; n = 2,620, Group 2; n = 2,851, Group3; n = 3,044, Group4; n = 2,766.

^§^: Sample size in each BMI-RMSE group Group1; n = 2,875, Group 2; n = 2,684, Group3; n = 3,123, Group4; n = 2,599.

^††^: Hypertension is adjusted for age, sex, alcohol, smoking habits, exercise, marriage, family history, medication use, dyslipidemia in 2009, diabetes mellitus in 2009, BMI, (for BF%-RMSE) BF%-slope, and (for BMI-RMSE) BMI-slope.

^§§^: Dyslipidemia is adjusted for age, sex, alcohol, smoking habits, exercise, marriage, family history, medication use, hypertension in 2009, diabetes mellitus in 2009, BMI, (for BF%-RMSE) BF%-slope, and (for BMI-RMSE) BMI-slope.

***: Diabetes Mellitus is adjusted for age, sex, alcohol, smoking habits, exercise, marriage, family history, medication use, hypertension in 2009, dyslipidemia in 2009, BMI, (for BF%-RMSE) BF%-slope and (for BMI-RMSE) BMI-slope.

To validate the results, we next performed a sensitivity analysis. As a substitute for BF%, we used FM, and the index of FM over height squared (FM [kg] / (height [m])^2^). As shown in S1 Table, overall patterns were similar in all models.

### BF%-RMSE vs. BMI-RMSE

We next compared our subjects by the four BMI-RMSE groups to the BF%-RMSE groups using multivariate logistic regression analyses (Table 3). There was no significant difference in the incidence of hypertension or dyslipidemia between the 4 groups. The BMI-RMSE Group 3 showed a non-significant decreased diabetes mellitus incidence (OR = 0.74; 95%CI = 0.54–1.00; *p* = 0.05), but there was no overall tendency. We also constructed two additional models adjusting for different subsets of the covariates, and odds ratios were similar in all models (S2 and S3 Tables).

### Stratification by gender

We re-performed the multivariate logistic regression analysis after stratifying by gender because the variability in BF%-RMSE differs by gender. The baseline characteristics of the participants are shown in Tables 4 and 5.

**Table 4:** Baseline male characteristics of participants.

variables	BF%-RMSE					*p* values
	Group 1 (*n* = 1,449)	Group 2 (*n* = 1,384)	Group 3 (*n* = 1,310)	Group 4 (*n* = 1,359)	Total (*n* = 5,502)	
	(0-25^th^ percentile)	(26-50^th^ percentile)	(51-75^th^ percentile)	(76-100^th^ percentile)		
Age, mean, years (SD)	52.8 (11.3)	53.0 (11.5)	52.3 (11.0)	51.2 (11.7)	52.4 (11.3)	**<0.01**
Alcohol abuse, *n*(%)	657 (45.3)	641 (46.3)	635 (48.5)	659 (48.5)	2,592 (47.1)	0.24
Smoking status						0.36
current smoker, *n*(%)	293 (20.2)	283 (20.4)	289 (22.1)	317 (23.3)	1,182 (21.5)	
ex-smoker, *n*(%)	573 (39.5)	550 (39.7)	528 (40.3)	516 (38.0)	2,167 (39.4)	
Exercise						0.10
everyday, *n*(%)	200 (13.8)	185 (13.4)	168 (12.8)	166 (12.2)	719 (13.1)	
3–5 days/week, *n*(%)	272 (18.8)	226 (16.3)	229 (17.5)	225 (16.6)	952 (17.3)	
1–2 days/week, *n*(%)	545 (37.6)	592 (42.8)	532 (40.6)	531 (39.1)	2,200 (40.0)	
less than 1 day/week, *n*(%)	432 (29.8)	381 (27.5)	381 (29.1)	437 (32.2)	1,631 (29.6)	
Married, *n*(%)	1,294 (89.3)	1,213 (87.6)	1,159 (88.5)	1,169 (86.0)	4,835 (87.9)	**0.03**
Family history of CVA, ACS*, *n*(%)	255 (17.6)	279 (20.2)	275 (21.0)	260 (19.1)	1,069 (19.4)	0.13
Medication use, *n*(%)	270(18.6)	257(18.6)	231(17.6)	234(17.2)	992(18.0)	0.71
Hypertension in 2009, *n*(%)	443 (30.6)	435 (31.4)	416 (31.8)	392 (28.8)	1,686 (30.6)	0.36
Dyslipidemia in 2009, *n*(%)	687 (47.4)	675 (48.8)	639 (48.8)	624 (45.9)	2,625 (47.7)	0.39
Diabetes Mellitus in 2009, *n*(%)	107 (7.4)	105 (7.6)	92 (7.0)	121 (8.9)	425 (7.7)	0.28
BMI, mean(SD)	23.5 (2.8)	23.5 (2.7)	23.8 (2.9)	24.4 (3.2)	23.8 (2.9)	**<0.01**
BF%, mean, %(SD)	21.1 (4.6)	21.3 (4.7)	21.9 (4.8)	23.0 (5.2)	21.8 (4.9)	**<0.01**
BF%-slope, mean(SD)	-0.21 (1.11)	-0.27 (1.17)	-0.30 (1.22)	-0.57 (1.62)	-0.33 (1.30)	**<0.01**

CVA, cerebrovascular accidents; ACS, acute coronary syndrome; BMI, body mass index; BF%, body fat percentage

*: Family history of CVA, ACS included family history of myocardial infarction, angina, stroke, brain hemorrhage, and subarachnoid hemorrhage in second degree relatives.

**Table 5:** Baseline female characteristics of participants.

variables	BF%-RMSE					*p* values
	Group 1 (*n* = 1,410)	Group 2 (*n* = 1,525)	Group 3 (*n* = 1,367)	Group 4 (*n* = 1,477)	Total (*n* = 5,779)	
	(0-25^th^ percentile)	(26-50^th^ percentile)	(51-75^th^ percentile)	(76-100^th^ percentile)		
Age, mean, years (SD)	50.0 (10.6)	50.6 (10.8)	50.6 (10.7)	49.9 (10.7)	50.3 (10.7)	0.21
Alcohol abuse, *n*(%)	546 (38.7)	608 (39.9)	552 (40.4)	569 (38.5)	2,275 (39.4)	0.70
Smoking status						0.90
current smoker, *n*(%)	94 (6.7)	92 (6.0)	91 (6.7)	102 (6.9)	379 (6.6)	
ex-smoker, *n*(%)	136 (9.6)	137 (9.0)	123 (9.0)	125 (8.5)	521 (9.0)	
Exercise						0.29
everyday, *n*(%)	154 (10.9)	171 (11.2)	150 (11.0)	140 (9.5)	615 (10.6)	
3–5 days/week, *n*(%)	266 (18.9)	269 (17.6)	225 (16.5)	266 (18.0)	1,026 (17.8)	
1–2 days/week, *n*(%)	510 (36.2)	551 (36.1)	515 (37.7)	509 (34.5)	2,085 (36.1)	
less than 1 day/week, *n*(%)	480 (34.0)	534 (35.0)	477 (34.9)	562 (38.1)	2,053 (35.5)	
Married, *n*(%)	891 (63.2)	999 (65.5)	858 (62.8)	885 (59.9)	3,633 (62.9)	**0.02**
Family history of CVA, ACS*, *n*(%)	332 (23.5)	364 (23.9)	320 (23.4)	343 (23.2)	1,359 (23.5)	0.98
Medication use, *n*(%)	122(8.7)	150(9.8)	144(10.5)	181(12.3)	597(10.3)	**0.01**
Hypertension in 2009, *n*(%)	192 (13.6)	261 (17.1)	233 (17.0)	281 (19.0)	967 (16.7)	**0.00**
Dyslipidemia in 2009, *n*(%)	459 (32.6)	490 (32.1)	463 (33.9)	538 (36.4)	1,950 (33.7)	0.06
Diabetes Mellitus in 2009, *n*(%)	36 (2.6)	35 (2.3)	31 (2.3)	50 (3.4)	152 (2.6)	0.20
BMI, mean(SD)	20.7 (2.9)	20.8 (2.8)	21.1 (3.0)	22.1 (3.4)	21.2 (3.0)	**<0.01**
BF%, mean, %(SD)	24.7 (5.4)	24.8 (5.4)	25.6 (5.6)	27.7 (6.4)	25.7 (5.8)	**<0.01**
BF%-slope, mean(SD)	-0.01 (1.14)	-0.05 (1.13)	-0.07 (1.22)	-0.26 (1.68)	-0.10 (1.32)	**<0.01**

CVA, cerebrovascular accidents; ACS, acute coronary syndrome; BMI, body mass index; BF%, body fat percentage

*: Family history of CVA, ACS included family history of myocardial infarction, angina, stroke, brain hemorrhage, and subarachnoid hemorrhage in second degree relatives.

Table 6 demonstrates the associations between BF%-RMSE and the newly diagnosed cardiovascular disease risk factors identified in the multivariate logistic regression analysis. Groups with a high BF%-RMSE showed a higher incidence of hypertension in males, but this tendency was not present in female subjects. For dyslipidemia, a significantly higher incidence was observed in Group 4 in female subjects (OR = 1.28; 95%CI = 1.02–1.62; *p* = 0.03), but this was not observed in male subjects. For diabetes mellitus, Group 4 showed a significantly lower incidence in male subjects (OR = 0.62; 95%CI = 0.40–0.94; *p* = 0.03), but there was no significant difference between groups in female subjects.

**Table 6:** Associations between BF%-RMSE and the newly diagnosed cardiovascular risk factors in the multivariate logistic regression analysis in each gender.

	Odds ratio (95% confidence interval)			
Male	Group 1 (*n* = 1,449)	Group 2 (*n* = 1,384)	Group 3 (*n* = 1,310)	Group 4 (*n* = 1,359)
	(0-25^th^ percentile)	(26-50^th^ percentile)	(51-75^th^ percentile)	(76-100^th^ percentile)
Hypertension^†^	Reference	1.21 (0.98–1.51)**	1.21 (0.97–1.51)**	1.24(0.99–1.54)**
Dyslipidemia^§^	Reference	1.06 (0.85–1.33)	0.80 (0.63–1.02)**	0.95(0.75–1.19)
Diabetes Mellitus^‡^	Reference	0.99 (0.68–1.45)	0.99 (0.68–1.45)	0.62(0.40–0.94)*
Female	Group 1 (*n* = 1,410)	Group 2 (*n* = 1,525)	Group 3 (*n* = 1,367)	Group 4 (*n* = 1,477)
	(0-25^th^ percentile)	(26-50^th^ percentile)	(51-75^th^ percentile)	(76-100^th^ percentile)
Hypertension^†^	Reference	0.80 (0.62–1.05)	0.88 (0.67–1.15)	1.10 (0.85–1.42)
Dyslipidemia^§^	Reference	1.19 (0.95–1.50)	1.07 (0.84–1.36)	1.28 (1.02–1.62)*
Diabetes Mellitus^‡^	Reference	1.10 (0.66–1.86)	0.87 (0.50–1.52)	0.78 (0.45–1.34)

*: p<0.05,

**: p<0.10

^†^: Hypertension is adjusted for age, alcohol, smoking habits, exercise, marriage, family history, medication use, dyslipidemia in 2009, diabetes mellitus in 2009, BMI, and BF%-slope.

^§^: Dyslipidemia is adjusted for age, alcohol, smoking habits, exercise, marriage, family history, medication use, hypertension in 2009, diabetes mellitus in 2009, BMI, and BF%-slope.

^‡^: Diabetes Mellitus is adjusted for age, alcohol, smoking habits, exercise, marriage, family history, medication use, hypertension in 2009, dyslipidemia in 2009, BMI, and BF%-slope.

## Discussion

In this study, we analyzed the association between BF% variability and cardiovascular risk factors. Surprisingly, a high BF% variability was associated with conflicting outcomes: a high incidence of hypertension and a low incidence of diabetes mellitus. These associations were clear in the male population, but not in women, after stratifying by gender. An association between BMI-RMSE and cardiovascular risk factors was not observed in our study, which is consistent with previous retrospective cohort studies [13].

To the best of our knowledge, this is the first paper to investigate the association of BF% variability with cardiovascular risk factors. BF% variability, not BMI variability, affected cardiovascular risks, which suggests a stronger impact of body proportion variability than body weight variability on cardiovascular risk.

Recent basic research studies suggest that adipose cell size is not static. The distribution in adipose cell size changes through the diverse situations in an individual’s life [46–48]. Therefore, body fat percentage (BF%) variability seems to be associated with dynamics in adipose cell size. As the turnover in white adipose tissue is extremely slow, body fat variability implies a repeated discharge and accumulation of fat in existing cells. Hypertrophy of adipocytes is associated with inflammatory changes via several cytokines [49–50], and thus repeated hypertrophy of adipocytes may represent a potential mechanism by which BF% variability affects cardiovascular risk.

Our results can provide a clear explanation of the contradicting outcomes between BMI variability and mortality. Some previous studies have shown an association between BMI variability and mortality [10–11], but this association was not observed in other studies [12–13]. BF% was not measured in these studies, but the subjects with a high BMI variability included in these studies may have included different percentages of subjects with a high BF% variability, and this heterogeneity could have led to the different outcomes. Indeed, a previous study showed that both body fat mass and lean mass dynamically change after weight loss and gain [51]. Additionally, subjects with a high BF% variability may have different later mortality rates based on the subject’s age, sex and other cardiovascular risk factors because of the differences in gender and the conflicting results regarding BF% variability and cardiovascular risk factors. Further studies with outcomes of cardiovascular events or mortality should be conducted.

Several limitations of this study should be acknowledged. First, our study population had relatively low percentage of overweight subjects. In our study, 20.4% of the study subjects were overweight (BMI≥25). This prevalence rate is similar to that reported in Japanese population studies [52–53], but is different from previous variability studies. Second, our subjects were limited to those who attended several medical check-up visits. These subjects are more conscious of their health. The prevalence of smokers and alcohol abusers in our subjects was lower than in those who received a medical check-up in 2005 but not in the following years (Table 1). Since smoking is known to affect body weight [54], this limits the generalizability of our work. However, it can be an advantage of our study, because our study participants have low risk of smoking cessation during follow-up. Third, our study lacked information on the reasons for the changes in body fat percentage and weight. We were unable to distinguish the effects of intentional diet from unintentional body fat changes. Fourth, the information on hypertension, dyslipidemia, and diabetes mellitus was partially obtained from self-reporting questionnaires. We used blood tests to supplement the limitations of self-reporting questionnaires, but this might not have been an ideal approach. Fifth, the RMSE can be influenced by outliners (due to illness, for example). However, outliers due to illness may be rare, because the medical check-up examinees are apparently healthy individuals. Lastly, since the current study is observational, clinical trials investigating the favorable effects of reducing BF% variability are needed to elucidate any direct causality. Nevertheless, the longitudinal nature of cohort studies enables the assessment of causal hypotheses, as exposure occurred prior to outcome [55].

In summary, we performed a retrospective cohort analysis using single-center medical check-up data to clarify the relationship between BF% and cardiovascular risk factors. High BF% fluctuation was associated with a high incidence of hypertension and a low incidence of diabetes mellitus. This relationship was not observed when repeating the same analyses using BMI variability. The identified relationship was maintained in male subjects, but not in female subjects. The effects of BF% variability on cardiovascular events were not investigated, but our results may partially explain the complicated outcomes regarding BMI variability and cardiovascular events in previous studies.

## Supporting information

S1 Table Sensitivity analysis using different parameters to describe body fat. (PDF)

S2 Table Associations between BF%-RMSE/BMI-RMSE and the newly diagnosed cardiovascular risk factors in logistic regression model 2. (PDF)

S3 Table Associations between BF%-RMSE/BMI-RMSE and the newly diagnosed cardiovascular risk factors in logistic regression model 3. (PDF)
