# Supplementary material for: Associations between body fat variability and later onset of cardiovascular disease risk factors
Source: PLoS One. 2017 Apr 3;12(4):e0175057. doi: 10.1371/journal.pone.0175057 (PMC5378370; doi:10.1371/journal.pone.0175057)
Supplement: S1 Table — (PDF) [file pone.0175057.s001.pdf]

S1 Table. Sensitivity analysis using different parameters to describe body fat

	Odds ratio (95% confidence interval)			
	Group 1 (0-25th percentile)	Group 2 (26-50th percentile)	Group 3 (51-75th percentile)	Group 4 (76-100th percentile)
BF%-RMSE†				
Hypertension††	Reference	0.99 (0.83-1.18)	1.02 (0.86-1.21)	**1.17**** **(0.99-1.39)**
Dyslipidemia§§	Reference	**1.15**** **(0.98-1.36)**	0.98 (0.83-1.16)	1.14 (0.97-1.35)
Diabetes Mellitus¶¶	Reference	1.00 (0.73-1.37)	0.91 (0.66-1.25)	**0.68*** **(0.49-0.96)**
FM-RMSE§				
Hypertension††	Reference	**0.82*** **(0.69-0.98)**	1.13 (0.95-1.33)	1.10 (0.93-1.31)
Dyslipidemia§§	Reference	**1.15**** **(0.98-1.35)**	1.00 (0.85-1.18)	1.10 (0.92-1.30)
Diabetes Mellitus¶¶	Reference	1.07 (0.78-1.47)	0.77 (0.55-1.08)	0.82 (0.59-1.13)
Fat Index-RMSE¶				
Hypertension††	Reference	0.89 (0.75-1.06)	1.11 (0.94-1.32)	1.15 (0.97-1.37)
Dyslipidemia§§	Reference	**1.15**** **(0.98-1.35)**	0.96 (0.81-1.13)	1.12 (0.94-1.32)
Diabetes Mellitus¶¶	Reference	1.03 (0.75-1.42)	0.86 (0.62-1.20)	**0.74**** **(0.53-1.03)**

*: $p<0.05$, **: $p<0.10$

†: Sample size in each BF%-RMSE group Group1; n=2,620, Group 2; n=2,851, Group3; n=3,044, Group4; n=2,766.

§: Sample size in each FM-RMSE group Group1; n=2821, Group 2; n=2820, Group3; n=2819, Group4; n=2821.

¶: The index of total body fat mass over height squared is named as "Fat Index" for convenience sake.

Sample size in each Fat Index-RMSE group Group1; n=2,821, Group 2; n=2,820, Group3; n=2,819, Group4; n=2,821.

††: Hypertension is adjusted for age, sex, alcohol, smoking habits, exercise, marriage, family history, medication use, dyslipidemia in 2009, diabetes mellitus in 2009, BMI, (for BF%-RMSE) BF%-slope, (for FM-RMSE) FM-slope, and (for Fat Index-RMSE) Fat Index-slope.

§§: Dyslipidemia is adjusted for age, sex, alcohol, smoking habits, exercise, marriage, family history, medication use, hypertension in 2009, diabetes mellitus in 2009, BMI, (for BF%-RMSE) BF%-slope, (for FM-RMSE) FM-slope, and (for Fat Index-RMSE) Fat Index-slope.

¶¶: Diabetes Mellitus is adjusted for age, sex, alcohol, smoking habits, exercise, marriage, family history, medication use, hypertension in 2009, dyslipidemia in 2009, BMI, (for BF%-RMSE) BF%-slope, (for FM-RMSE) FM-slope, and (for Fat Index-RMSE) Fat Index-slope.
