# Supplementary material for: Associations between body fat variability and later onset of cardiovascular disease risk factors
Source: PLoS One. 2017 Apr 3;12(4):e0175057. doi: 10.1371/journal.pone.0175057 (PMC5378370; doi:10.1371/journal.pone.0175057)
Supplement: S3 Table — (PDF) [file pone.0175057.s003.pdf]

S3 Table. Associations between BF%-RMSE/BMI-RMSE and the newly diagnosed cardiovascular risk factors in logistic regression model 3

	Odds ratio (95% confidence interval)			
	Group 1 (0-25th percentile)	Group 2 (26-50th percentile)	Group 3 (51-75th percentile)	Group 4 (76-100th percentile)
BF%-RMSE†				
Hypertension††	Reference	1.00 (0.84-1.19)	1.03 (0.88-1.22)	**1.18**** **(1.00-1.40)**
Dyslipidemia§§	Reference	1.15 (0.97-1.35)	0.99 (0.84-1.17)	1.14 (0.96-1.35)
Diabetes Mellitus***	Reference	1.02 (0.75-1.40)	0.93 (0.68-1.27)	**0.70*** **(0.50-0.98)**
BMI-RMSE§				
Hypertension††	Reference	1.12 (0.95-1.33)	1.13 (0.97-1.33)	1.11 (0.93-1.32)
Dyslipidemia§§	Reference	0.94 (0.80-1.11)	1.04 (0.89-1.21)	1.01 (0.85-1.19)
Diabetes Mellitus***	Reference	0.87 (0.63-1.19)	**0.76**** **(0.55-1.03)**	0.82 (0.60-1.12)

*: p<0.05, **: p<0.10

†: Sample size in each BF%-RMSE group Group1; n=2,620, Group 2; n=2,851, Group3; n=3,044, Group4; n=2,766.

§: Sample size in each BMI-RMSE group Group1; n=2,875, Group 2; n=2,684, Group3; n=3,123, Group4; n=2,599.

††: Hypertension is adjusted for age, sex, dyslipidemia in 2009, diabetes mellitus in 2009, BMI, (for BF%-RMSE) BF%-slope, and (for BMI-RMSE) BMI-slope.

§§: Dyslipidemia is adjusted for age, sex, hypertension in 2009, diabetes mellitus in 2009, BMI, (for BF%-RMSE) BF%-slope, and (for BMI-RMSE) BMI-slope.

***: Diabetes Mellitus is adjusted for age, sex, hypertension in 2009, dyslipidemia in 2009, BMI, (for BF%-RMSE) BF%-slope, and (for BMI-RMSE) BMI-slope.
